# Hearing results following endoscopic type I tympanoplasty in medium and large perforations

**DOI:** 10.1016/j.bjorl.2024.101509

**Published:** 2024-09-10

**Authors:** Lihua Yue, Xiao Liu, Hongyan Liu, Haina Ma

**Affiliations:** Zhejiang University School of Medicine, The Second Affiliated Hospital, Department of Otolaryngology, Hangzhou, China

**Keywords:** Type I tympanoplasty, Endoscopic, Hearing, Tympanic membrane perforation, Chronic otitis media

## Abstract

**Objectives:**

To clarify the hearing outcomes after endoscopic type I tympanoplasty for medium and large perforations due to chronic otitis media.

**Methods:**

We examined the clinical records of patients who underwent endoscopic type I tympanoplasty for medium and large perforations of the eardrum resulting from chronic otitis media between January 2019 and December 2021. We analyzed the changes in hearing pre- and post-operation in patients with healed eardrums and assessed the impact of tympanosclerosis on hearing. Patients with incomplete follow-up data, middle ear cholesteatoma, stapes fixation, severe lesions in the tympanic antrum and mastoid necessitating mastoidectomy and/or ossicular chain reconstruction were excluded.

**Results:**

A total of 156 patients underwent analysis for audiological outcomes. Among them, 63 had medium tympanic membrane perforations, with 18 cases showing calcification of the tympanic membrane and 20 cases with calcification in the tympanic cavity. Additionally, 93 cases had large tympanic membrane perforations, with 25 cases showing tympanic membrane calcification and 32 cases with tympanic cavity calcification. Prior to surgery, the Air Conduction threshold (AC) in the large perforation group was higher than in the medium perforation group, particularly at low frequencies, measuring (47.4 ± 13.3 dB) and (41.2 ± 14.7 dB), respectively (*p*-value < 0.05). Following surgery, both groups experienced an improvement in AC, measuring (33.6 ± 13.9 dB) and (32.6 ± 12.8 dB), respectively, with no significant difference noted (*p*-value > 0.05). There was no significant change in Bone Conduction threshold (BC) before and after surgery in either the large or medium perforation groups (all *p*-values > 0.05). Except for 4000 Hz an increase, bone conduction did not increase post-surgery, instead showing further improved. Pre-surgery, the Air-Bone Gap (ABG) in the large and medium perforation groups was (27.7 ± 8.5 dB) and (21.8 ± 8.3 dB), respectively, mainly affecting low frequencies, with a statistically significant difference noted (*p*-value < 0.05). Following surgery, ABG in both groups improved to (16.3 ± 7.6 dB) and (15.7 ± 8.4 dB), respectively, with no significant difference observed (*p*-value > 0.05). There was no significant difference in hearing pre-surgery among the groups with No calcification (No), Tympanic Membrane Calcification (TM), and Tympanic Cavity Calcification (TC). However, TC significantly impacted low frequency (250–500 Hz) AC and ABG. The differences in AC and ABG pre-surgery between TC and No group, and TC and TM group (at 250–500 Hz) were statistically significant (all *p*-values < 0.05). Preoperative ABG in TM group was better than in No group and TC group, suggesting minimal impact of tympanic membrane calcification on hearing. No interaction was observed between tympanic membrane perforation size and tympanosclerosis on hearing. Post-surgery, both large and medium tympanic membrane perforation groups, regardless of tympanosclerosis presence, showed good AC and ABG, with no statistically significant difference in △ABG (all *p*-values > 0.05).

**Conclusion:**

Preoperative AC and ABG were increase in cases of large tympanic membrane perforations and medium tympanic membrane perforations with tympanic cavity calcification. Surgical intervention led to more significant hearing improvement in these patients. However, irrespective of tympanic membrane perforation size and the presence of tympanosclerosis, as long as the ossicular chain remains intact and functional, postoperative AC and ABG outcomes are satisfactory. Endoscopic type I tympanoplasty proves effective in achieving improved hearing outcomes for patients with medium to large tympanic membrane perforations and tympanosclerosis, provided there is no ossicle chain fixation.

**Level of evidence:**

Level 4.

## Highlights

• Chronic otitis media leads to tympanic membrane perforation and hearing impairment.
• Endoscopic ear surgery has the advantages of minimally invasive.
• Endoscopic type I tympanoplasty yield satisfactory hearing results in patients with medium to large tympanic membrane perforations.

## Introduction

Chronic otitis media over time leads to tympanic membrane perforation, recurrent purulent otorrhea, and hearing impairment. Some patients may not experience purulent otorrhea episodes but suffer from residual tympanic membrane perforation, resulting in secondary tympanosclerosis, significant hearing loss, and an inability to swim.^1^ With improved living standards and increased life expectancy, the inability to wear hearing aids due to otitis media or tympanic membrane perforation becomes a significant concern. Transcanal endoscopic ear surgery has advanced, allowing for most tympanoplasties to be performed without postauricular skin incisions and external auditory canalplasty. This approach facilitates exploration of the auditory ossicle chain without bone grinding, enabling close observation of the tympanic cavity structure, including the posterior tympanic cavity and the tympanic isthmus passage.^2^ Even mild inflammation in the epitympanum, tympanic antrum, or mastoid can be addressed by removing lesions in the attic and tympanic isthmus, improving ventilation and drainage in the middle ear, and achieving favorable outcomes.^3^, ^4^ The use of cartilage–perichondrium grafts provides firmness, resistance to negative pressure, ease of manipulation with one hand, and is particularly suitable for repairing larger tympanic membrane perforations and in patients with poor Eustachian tube function.^5^ Medium and large tympanic membrane perforations are associated with a prolonged disease course, more severe tympanic cavity lesions, and significant hearing loss compared to small perforations. Patients with medium and large perforations often have high expectations for hearing improvement post-surgery.^6^ This study reviews the clinical outcomes of patients with medium and large tympanic membrane perforations caused by chronic otitis media who underwent endoscopic type I tympanoplasty, comparing preoperative and postoperative hearing changes.

## Methods

With approval from the hospital ethics committee, we retrospectively reviewed the clinical data of patients with medium and large perforations of the tympanic membrane caused by chronic otitis media. These patients underwent surgery performed by the same attending surgeon between January 2019 and December 2021. The ears were required to be dry for at least one month before surgery. Prior to the operation, video otoendoscopy, pure-tone audiometry, and temporal bone CT scans were conducted. The tympanic membrane was divided into four quadrants based on the position of the handle of the malleus, with each quadrant representing 25% of the membrane. Perforations were classified as medium if they spanned 25%–50% of the membrane and large if they exceeded 50%. Tympanosclerosis was categorized as follows: No (No tympanosclerosis), TM (Tympanic Membrane Calcification), and TC (Tympanic Cavity Calcification, including calcification and scar adhesions around the auditory ossicles and the tympanic isthmus). Patients with incomplete follow-up data, middle ear cholesteatoma, stapes fixation, or severe lesions in the tympanic antrum and mastoid requiring mastoidectomy and/or ossicular chain reconstruction were excluded.

### Surgical procedure

All patients underwent endoscopic type I tympanoplasty under general anesthesia, using a rigid 0° endoscope, 14 cm long and 3 mm in diameter or 18 cm long and 4 mm in diameter. A lateral canal incision was made approximately 5–10 mm from the annulus. The tympanomeatal flap was then elevated and detached from the malleus handle to provide complete visualization of the middle ear cavity. The middle ear cavity and auditory ossicle chain were examined. Any tympanosclerotic plaques and scar adhesions around the tympanic membrane, auditory ossicles, and anterior tympanic isthmus were removed. Tympanic membrane perforations were repaired using tragus cartilage-perichondrium grafts. Healing of the tympanic membrane and hearing outcomes were assessed at 3 weeks, 3 months, and 6 months post-surgery. Follow-up duration was at least 6 months.

### Audiological evaluation

All patients underwent preoperative and postoperative pure-tone audiometry. Hearing assessments conducted within 1 month before surgery and at least 3 months after surgery were used as statistical indicators. The average hearing thresholds for Air Conduction (AC), Bone Conduction (BC), and Air-Bone Gap (ABG) at frequencies of 500 Hz, 1000 Hz, 2000 Hz, and 4000 Hz (500–4000 Hz) were routinely recorded. Given previous findings indicating worse in low-frequency air conduction in tympanic membrane perforations,^7^ we also included 250 Hz in the analysis (250–4000 Hz). An increase in bone conduction threshold at each frequency before and after surgery exceeding 10 dB was considered indicative of sensorineural hearing loss. The changes in hearing at frequencies of 250–4000 Hz pre- and post-operation in patients with medium and large perforations were anaylsed.

### Statistical analysis

Audiological data were analyzed using SPSS 26.0 statistical software. Non-parametric data between groups were compared using the Mann–Whitney *U* or Kruskal–Wallis Test. Categorical data were analyzed using the Chi-Square test or exact probability method. A *p*-value < 0.05 was considered statistically significant.

## Results

A total of 201 patients were initially reviewed, with 26 patients lost to follow-up and 19 cases having unhealed tympanic membranes (7 cases of medium perforation and 12 cases of large perforation). Ultimately, 156 cases were included in the audiological results statistical analysis. Among them, 56 were males and 100 were females, with 87 left ears and 69 right ears, and an average age of 42 years (range: 20–71 years). There were 63 cases of medium perforation of the tympanic membrane, including 18 cases with TM and 20 cases with TC. Additionally, there were 93 cases of large perforation of the tympanic membrane, including 25 cases with TM and 32 cases with TC. There were no significant differences in age, gender, affected side, or tympanic membrane and tympanic cavity lesions between the two groups of patients (Table 1). The graft success rates in the medium perforation of the tympanic membrane and the large perforation of the tympanic membrane were 90% (63 out of 70 patients) and 89% (93 out of 105 patients), respectively (*p* > 0.05).

**Table 1:** Demographic characteristic of the patients.

Variable	Participants, n (%)			*p*-Value^a^
	Perforation size			
	Medium (n = 63)	Large (n = 93)	Overall (n = 156)	
Age, median (range), y	43 (20–71)	42 (21–69)	42 (20–71)	
30	14 (22.2)	15 (16.1)	29 (18.6)	0.60
30‒39	16 (25.4)	25 (26.9)	41 (26.3)	
40‒49	9 (14.3)	22 (23.7)	31 (19.9)	
50‒59	18 (28.6)	24 (25.81)	42 (26.9)	
>59	6 (9.5)	7 (7.5)	13 (8.3)	
Sex				
Male	26 (41.3)	30 (32.3)	56 (35.9)	0.25
Female	37 (58.7)	63 (67.7)	100 (64.1)	
Side				
Left	33 (52.4)	54 (58.1)	87 (55.8)	0.48
Right	30 (47.6)	39 (41.9)	69 (44.2)	
Surgical exploration				
No	25 (39.7)	36 (38.7)	61 (39.1)	0.94
TM	18 (28.6)	25 (26.9)	43 (27.6)	
TC	20 (31.8)	32 (34.4)	52 (33.3)	

No, No tympanosclerosis; TM, Tympanic Membrane Calcification; TC, Tympanic Cavity Calcification.

a *p*-Values are from Person χ^2^ test.

**Figure 1:**
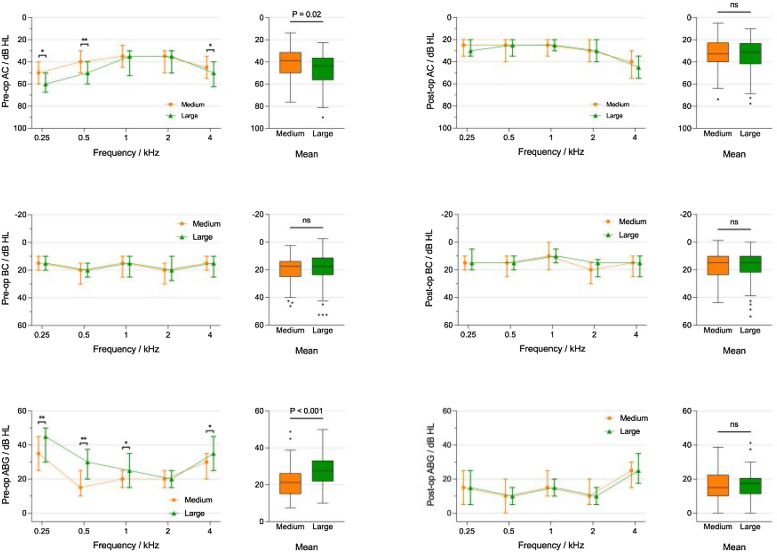
The impact of tympanic membrane perforation size on hearing.

The impact of tympanic membrane perforation size on hearing was assessed. Before surgery, the Air Conduction (AC) threshold of large perforations was higher than that of medium perforations, particularly at low frequencies, measuring (47.4 ± 13.3 dB) and (41.2 ± 14.7 dB), respectively. This difference was statistically significant (*p*-value < 0.05). Following surgery, the AC of both groups improved, measuring (33.6 ± 13.9 dB) and (32.6 ± 12.8 dB), respectively, with no statistically significant difference (*p*-value > 0.05). There were no significant differences in Bone Conduction (BC) between large and medium perforations before and after surgery (all *p*-values > 0.05), measuring (19.70 ± 11.96, 19.42 ± 9.92, and 17.23 ± 10.65, 16.87 ± 9.72), respectively. Apart from 4000 Hz an increase, there was no increase in bone conduction after surgery; instead, there was further improved. The Air-Bone Gap (ABG) before surgery for large and medium perforations was (27.7 ± 8.5 dB) and (21.8 ± 8.3 dB), respectively, with a statistically significant difference (*p*-value < 0.05). Following surgery, the ABG of both groups improved, measuring (16.3 ± 7.6 dB) and (15.7 ± 8.4 dB), respectively, with no statistically significant difference (*p*-value > 0.05) (Table 2 and Fig. 1).

**Table 2:** The changes in hearing pre- and post-operation in patients with medium and large perforations.

		Medium (n = 63)		Large (n = 93)		Independent-samples Mann–Whitney *U* test	
		Mean	SD	Mean	SD	Test statistic	*p*-Value
PreAC	250 Hz	52.4603	15.39462	59.3548	15.04027	2.8	0.005
	500 Hz	41.9841	14.2427	51.2903	16.3505	3.615	0
	1000 Hz	37.381	14.99616	42.5806	16.51108	0.1708	0.088
	2000 Hz	40.2381	15.33046	42.3118	16.11034	0.684	0.494
	4000 Hz	45.0794	16.61936	53.2796	18.98934	2.486	0.013
	Mean	41.1706	13.31397	47.3656	14.72283	2.331	0.02
PreBC	250 Hz	17.381	11.70391	16.5054	11.48688	−0.287	0.774
	500 Hz	22.0635	10.53546	21.2903	11.2265	−0.593	0.553
	1000 Hz	17.381	10.99329	17.4731	12.5244	−0.199	0.842
	2000 Hz	21.8254	12.32299	21.5591	14.16092	−0.372	0.71
	4000 Hz	16.4286	12.16249	18.4946	15.8763	0.292	0.771
	Mean	19.4246	9.92112	19.7043	11.9561	−0.454	0.65
PreABG	250 Hz	35.0794	12.96683	42.8495	13.23693	3.507	0
	500 Hz	19.9206	13.05979	30	13.79193	4.552	0
	1000 Hz	20	11.91367	25.1075	12.06816	2.694	0.007
	2000 Hz	18.4127	9.28168	20.7527	10.05275	1.336	0.182
	4000 Hz	28.6508	10.63342	34.7849	12.5307	3.427	0.001
	Mean	21.746	8.34772	27.6613	8.50636	4.207	0
PostAC	250 Hz	29.7619	14.40846	30.4301	13.32405	0.47	0.638
	500 Hz	29.0476	13.55565	27.6882	13.74376	−0.841	0.4
	1000 Hz	28.254	13.59432	28.1183	13.82959	−0.144	0.885
	2000 Hz	31.5873	13.73204	33.0645	16.11832	0.082	0.935
	4000 Hz	41.4286	17.58456	45.3763	18.97133	1.272	0.203
	Mean	32.5794	12.79466	33.5618	13.99722	0.036	0.971
PostBC	250 Hz	14.2063	8.85394	14.3548	9.09568	−0.011	0.991
	500 Hz	17.2222	9.53676	16.3441	9.53084	−0.721	0.471
	1000 Hz	11.3492	10.55731	11.828	11.32007	0.026	0.98
	2000 Hz	20	10.8509	21.1828	13.21638	0.007	0.994
	4000 Hz	18.8889	14.35282	19.5699	15.1201	0.343	0.732
	Mean	16.8651	9.7185	17.2312	10.64791	−0.029	0.977
PostABG	250 Hz	15.5556	13.10968	16.0753	12.5726	0.361	0.718
	500 Hz	11.8254	11.44065	11.3441	10.03092	0.04	0.968
	1000 Hz	16.9048	10.37322	16.2903	9.40927	−0.403	0.687
	2000 Hz	11.5873	8.07352	11.8817	7.58696	0.259	0.795
	4000 Hz	22.5397	11.4959	25.8065	11.01605	1.899	0.058
	Mean	15.7143	8.4114	16.3306	7.59855	0.345	0.73

**Figure 2:**
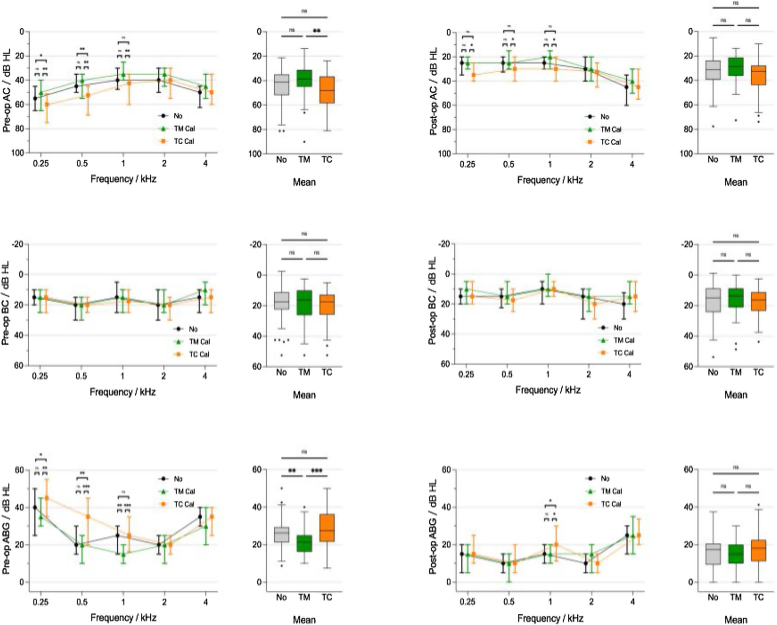
The effect of tympanosclerosis on hearing.

The effect of tympanosclerosis on hearing: There was no significant difference in preoperative hearing among the three groups: No (No tympanosclerosis), TM (Tympanic Membrane Calcification), and TC (Tympanic Cavity Calcification). However, the TC group had a significant impact on low-frequency (250–500 Hz) AC and ABG. Statistically significant differences in AC and ABG (250–500 Hz) before surgery were observed between the TC group and the No group and between the TC group and the TM group (*p*-values were all < 0.05). The ABG of the TM group was better than that of the No group and the TC group before surgery. Tympanic membrane calcification had little effect on hearing (Fig. 2).

**Figure 3:**
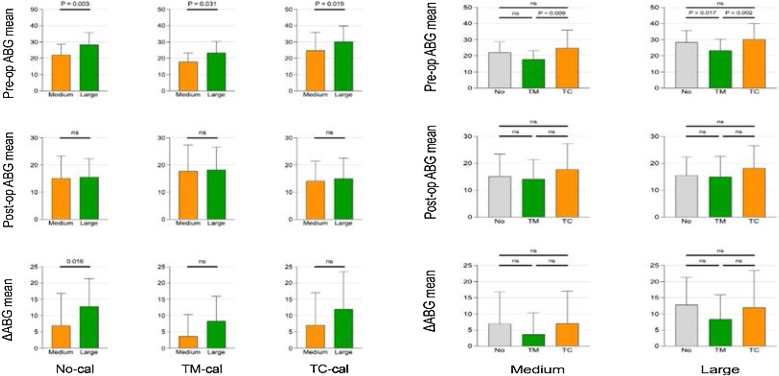
Interaction between the size of tympanic membrane perforation and tympanosclerosis.

Interaction between the size of tympanic membrane perforation and tympanosclerosis: regardless of the presence of TM or TC, the ABG of the large perforation group before surgery was greater than that of the medium perforation group (*p*-values were all < 0.05). In cases of medium perforation of the tympanic membrane, the ABG of the TC group before surgery was greater than that of the TM group (*p*-value < 0.05); in cases of large perforation of the tympanic membrane, the ABG of the TC group and the No group before surgery was greater than that of the TM group (*p*-values were all < 0.05). Regardless of large or medium perforations of the tympanic membrane and the presence or absence of tympanosclerosis, AC and ABG after surgery were both good, and the difference in △ABG was not statistically significant (*p*-values were all > 0.05) (Fig. 3).

Taste impairment occurred in 5 cases, all of which recovered 6 months after surgery. One case of delayed facial paralysis occurred after surgery, and recovery was observed 3 months post-surgery. No serious sensorineural hearing loss occurred after surgery.

## Discussion

With the development of endoscopic ear surgery, endoscopic tympanoplasty has become increasingly popular by providing a wide angle of view and illumination close to the TM. Several studies have shown that endoscopic tympanoplasty can achieve the same effect as microscopic surgery.^8^, ^9^, ^10^ Healing of the tympanic membrane and improvement of hearing are manifestations of the success of tympanoplasty. Literature shows that whether it is microscopic or endoscopic surgery, the healing rate of tympanic membrane after tympanoplasty is mostly around 90%, and patient age, size of tympanic membrane perforation, contralateral ear condition, and surgeon experience may be factors affecting the success of the surgery.^11^ All the patients in this study underwent the surgery completed by the same attending doctor. There were no differences in age, gender, affected side, and tympanic membrane or tympanic cavity lesions between the two groups of patients. There were 19 cases of unhealed tympanic membranes, 7 cases of medium perforation of the tympanic membrane, and 12 cases of large perforation of the tympanic membrane. There was no significant difference in the healing rate of the tympanic membranes between the two groups.

The size of tympanic membrane perforation correlates with the extent of conductive hearing loss when the ossicular chain remains intact and flexible. Generally, larger perforations result in greater conductive hearing loss compared to smaller defects.^12^, ^13^ In a study by Kolluru et al.,^14^ involving 155 ears of 151 patients with tympanic membrane perforations, it was observed that, except for 2000 Hz, the size of the perforation exhibited a linear correlation with conductive hearing loss, while the location of the perforation had minimal impact on the degree of hearing loss. Similarly, Bevis et al.^15^ conducted a cadaveric study on the middle ear’s conducting function using six fresh specimens and found that larger tympanic membrane perforations led to worsened conducting function, with posterior perforations having a more pronounced effect than anterior ones. Conversely, Saliba et al.^16^ suggested that while the degree of hearing loss in cases of small perforations might be influenced by the site of the perforation, for medium and large perforations involving more than two quadrants, the location of the perforation had negligible effect on hearing. In our study, all patients had medium and large perforations of the tympanic membrane due to chronic otitis media, consistent with existing literature. The AC and ABG of large perforations were higher than those of medium perforations before surgery, with a more significant worse noted in low frequencies. However, post-surgery, the AC and ABG of both patient groups improved, with no significant difference observed. This suggests that larger tympanic membrane perforations are associated with more severe conductive hearing loss, emphasizing the importance of prompt tympanic membrane repair to improve hearing. Utilizing tragal cartilage with perichondrium as a repair material for larger perforations was advantageous due to its location and accessibility. When the thickness is less than 0.5 mm, it does not impede the transmission of sound waves. All patients in our study achieved favorable outcomes after surgery.

Long-term recurrent chronic otitis media not only results in medium or large perforations of the tympanic membrane but also induces lesions in the tympanic cavity. These lesions, such as tympanosclerosis, scars, and adhesions around the auditory ossicles or the tympanic isthmus, can adversely affect hearing and lead to a significant air-bone conduction gap. Tympanosclerosis is characterized by hyaline degeneration and calcareous deposits in the tympanic membrane and middle ear cavity, impairing the mobility of the ossicular chain and hindering the healing of the tympanic membrane.^17^, ^18^ The surgical management of tympanosclerosis with tympanic membrane perforation has long been a subject of debate. According to the Wielinga–Kerr classification, there are four types of tympanosclerosis.^19^ Some researchers advocate for the extensive removal of sclerosis plaques around the auditory ossicles in the initial stage to facilitate ossicular mobility and improve hearing.^20^ Conversely, others oppose this approach, fearing that manipulation of the auditory ossicles may lead to sensorineural deafness and increase the risk of chronic otitis media recurrence. They suggest removing the fixed hammer and incus to reconstruct the ossicular chain or performing staged fenestra surgery on the stapes.^21^, ^22^ In a nine-year study, no stapes re-fixation was observed, and no differences were found in the long-term hearing outcomes between the two groups. Additionally, no cases of sensorineural deafness, tinnitus, or dizziness occurred after surgery.^23^, ^24^ This study included 95 cases of tympanosclerosis, excluding patients undergoing ossicular chain reconstruction or a second session for stapedectomy. Preoperative thin slice temporal bone CT scans were routinely conducted to rule out middle ear cholesteatoma. Temporal bone CT scans typically reveal the absorption and destruction of attic bone and ossicles with cholesteatoma, while tympanosclerosis may manifest as dot or slice-shaped high-density shadows, with no evident destruction of attic bone and ossicles.^25^ In this study, there was no notable variance in the average preoperative hearing thresholds among the No, TC, and TM groups. However, TC significantly impacted the low-frequency Air Conduction hearing threshold (AC) and the Air-Bone Conduction difference threshold (ABG). Specifically, preoperative AC and ABG (250–500) showed statistically significant differences between TC and No, as well as between TC and TM groups. During surgery, efforts were made to remove sclerosis plaques, scars, and adhesions that impede the healing of the tympanic membrane and the mobility of the auditory ossicles. Common factors affecting malleus movement included calcification of the anterior malleolar ligament and sclerosis around the malleus handle. Caution was exercised to avoid damaging the horizontal segment of the facial nerve while removing sclerosis plaques around the stapes. In some cases, even in the absence of sclerosis, anterior tympanic isthmus scars around the stapes and adhesions could affect auditory ossicle mobility, resulting in a significant air-bone gap. Therefore, during surgery, efforts were made to remove middle ear cavity scars and adhesions as much as possible to enhance middle ear ventilation and drainage. Tympanic membrane calcification had minimal impact on hearing, but calcium deposits on the perforation edge hindered tympanic membrane healing and were thus removed when feasible. There was no significant change in Bone Conduction threshold (BC) before and after surgery in either the large or medium perforation groups (all *p*-values > 0.05). Post-surgery, both large and medium tympanic membrane perforation groups, regardless of tympanosclerosis presence, showed good AC and ABG, with no statistically significant difference in △ABG (all *p*-values > 0.05). No serious sensorineural hearing loss occurred after surgery.

In this study, no interaction was observed between the size of the tympanic membrane perforation and tympanosclerosis. Regardless of whether the perforation was large or medium, and irrespective of the presence or absence of tympanosclerosis, postoperative AC and ABG outcomes were favorable, with no instances of severe sensorineural deafness noted. This finding may be attributed to the study’s patient selection criteria, which excluded individuals with fixed auditory ossicle chains and those requiring auditory ossicle chain reconstruction. For patients with medium or large tympanic membrane perforations and an ABG of 30 dB or more, it is recommended to perform ear canal skin flap surgery to explore the cavity and assess the presence of tympanosclerosis or lesions, as well as the mobility of the auditory ossicle chain. In cases of moderate to severe mixed hearing loss accompanied by tympanosclerosis, after lesion removal, intact and mobile auditory ossicle chains, and improved air conduction post-surgery, bone conduction may also show improvement. Possible explanations include inflammatory lesions in the tympanic cavity affecting the inner ear via the round window membrane, sclerosis lesions affecting auditory ossicle chain mobility, or loss of bone-conducted sound energy due to perforated tympanic membrane.^26^, ^27^ In our results, except 4000 Hz an increase 2 dB in medium perforation and 1 dB in large perforation, there was a little reduction in bone conduction after surgery. Therefore, even in the absence of purulent otorrhea, long-term medium or large tympanic membrane perforations may be accompanied by tympanosclerosis, leading to severe conductive hearing loss or mixed hearing loss. Timely tympanoplasty remains crucial for improving hearing in such cases.

As the follow-up period extends, certain patients may encounter complications such as myringitis, recurrent otitis media, re-perforation of the tympanic membrane, or hearing loss. Generally, longer follow-up durations provide more reliable data. The American Academy of Otolaryngology–Head and Neck Surgery recommends a minimum follow-up period of 1 year after middle ear surgery.^28^ However, conducting long-term follow-up poses challenges. Aabenhus et al.^29^ conducted a prospective follow-up of 1367 cases of tympanoplasty over 3–12 months, with a follow-up loss rate of 47.5%. They observed that most changes in AC following type I tympanoplasty were less than 10 dB at 3 and 12 months postoperatively. Their findings suggested that hearing outcomes at 3 months post-surgery could effectively represent those at 12 months post-surgery. In our analysis, we focused on hearing outcomes at least 3 months post-surgery, revealing significant improvements. However, long-term follow-up remains necessary to monitor changes in hearing, otitis media recurrence, re-perforation of the tympanic membrane, or secondary middle ear cholesteatoma.

Several limitations and shortcomings of our study are worth noting. Firstly, it employed a retrospective design, had a limited number of cases, Secondly, our study did not include speech recognition rate tests, which should be emphasized in future research. Larger with extended follow-up periods and multi-center participation are warranted.

## Conclusion

In conclusion, we believe that for individuals with chronic otitis media accompanied by medium or large perforations of the tympanic membrane, and even those with tympanosclerosis, timely tympanoplasty may lead to improved hearing outcomes, provided the auditory ossicle chain remains intact and mobile after the removal of sclerotic lesions, and postoperative AC and ABG are satisfactory. Even in cases of severe conductive or mixed hearing loss, tympanoplasty performed promptly can potentially enhance hearing. Notably, endoscopic type I tympanoplasty appears to yield satisfactory hearing results in patients with medium or large perforations of the tympanic membrane and tympanosclerosis without ossicle chain fixation.

## Funding and acknowledgments

This work was supported by Zhejiang Province Medical and Health Science and Technology Project (2023KY756).

## Conflicts of interest

The authors declare no conflicts of interest.
